# Current status of antimicrobial resistance in Indian healthcare system: combating antimicrobial resistance with precision medicine

**DOI:** 10.3389/frabi.2026.1632790

**Published:** 2026-01-28

**Authors:** Ashish Shinde, Athira Mohan, Vani Mahathi Bulusu, Poonam Soni, Jitendra Singh, Sagar Khadanga, Ankur Joshi, Poongothai Venkatachalapathy, Rupinder Kaur Kanwar, Sonal Sekhar Miraj, Murali Munisamy

**Affiliations:** 1 Department of Translational Medicine, All India Institute of Medical Sciences, Bhopal, India; 2 Department of General Medicine, All India Institute of Medical Sciences, Bhopal, United States; 3 Department of Community and Family Medicine, All India Institute of Medical Sciences, Bhopal, India; 4 Department of Pharmaceutical Sciences, College of Pharmacy and Pharmaceutical Sciences, Washington State University, Spokane, Washington, DC, United States; 5 Department of Pharmacy Practice, Manipal College of Pharmaceutical Sciences, Manipal Academy of Higher Education, Manipal, India

**Keywords:** antimicrobial resistance, AMR, carbapenem-resistant *Enterobacterales*, CRE, multi-drug-resistant gram-negative bacteria, MDR-GNB, *Acinetobacter baumanii*

## Abstract

This review provides a unique perspective by integrating antimicrobial resistance (AMR) data from Indian healthcare, with a particular emphasis on outpatient settings that are often overlooked in existing literature. Unlike previous reviews that primarily focus on hospital-acquired infections, this article explores the community dimension of AMR and its implications for public health. Furthermore, it introduces an innovative framework linking AMR mitigation strategies with precision medicine approaches, including pharmacogenomics, metabolomics, proteomics, and transcriptomics. By combining multi-omics insights with national surveillance data and stewardship initiatives, this review highlights the translational potential of personalized antimicrobial therapy tailored to the Indian healthcare ecosystem. This integrated perspective offers a novel direction for AMR research and policy, bridging the gap between genomic science and clinical application in resource-limited settings.

## 1 Introduction

**Figure 1:**
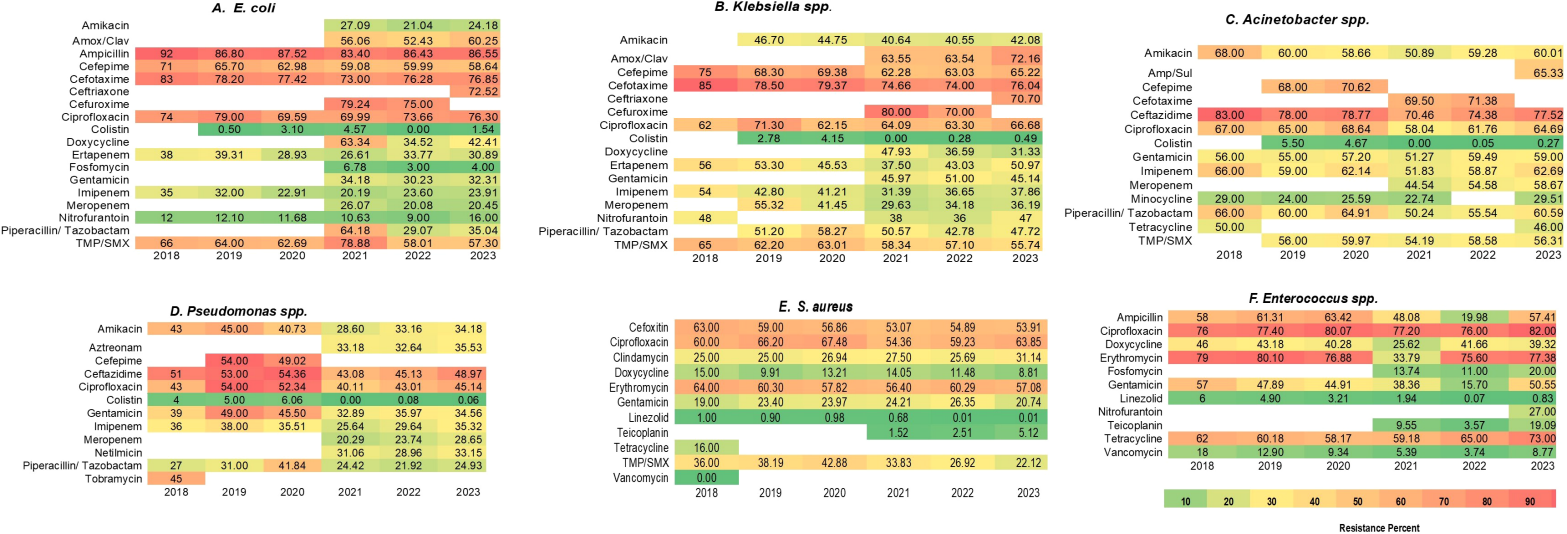
**(A–F)** Heatmap showing year wise trends of antimicrobial resistance in key pathogens data extracted from NARS-Net annual reports 2018-23.

**Figure 2:**
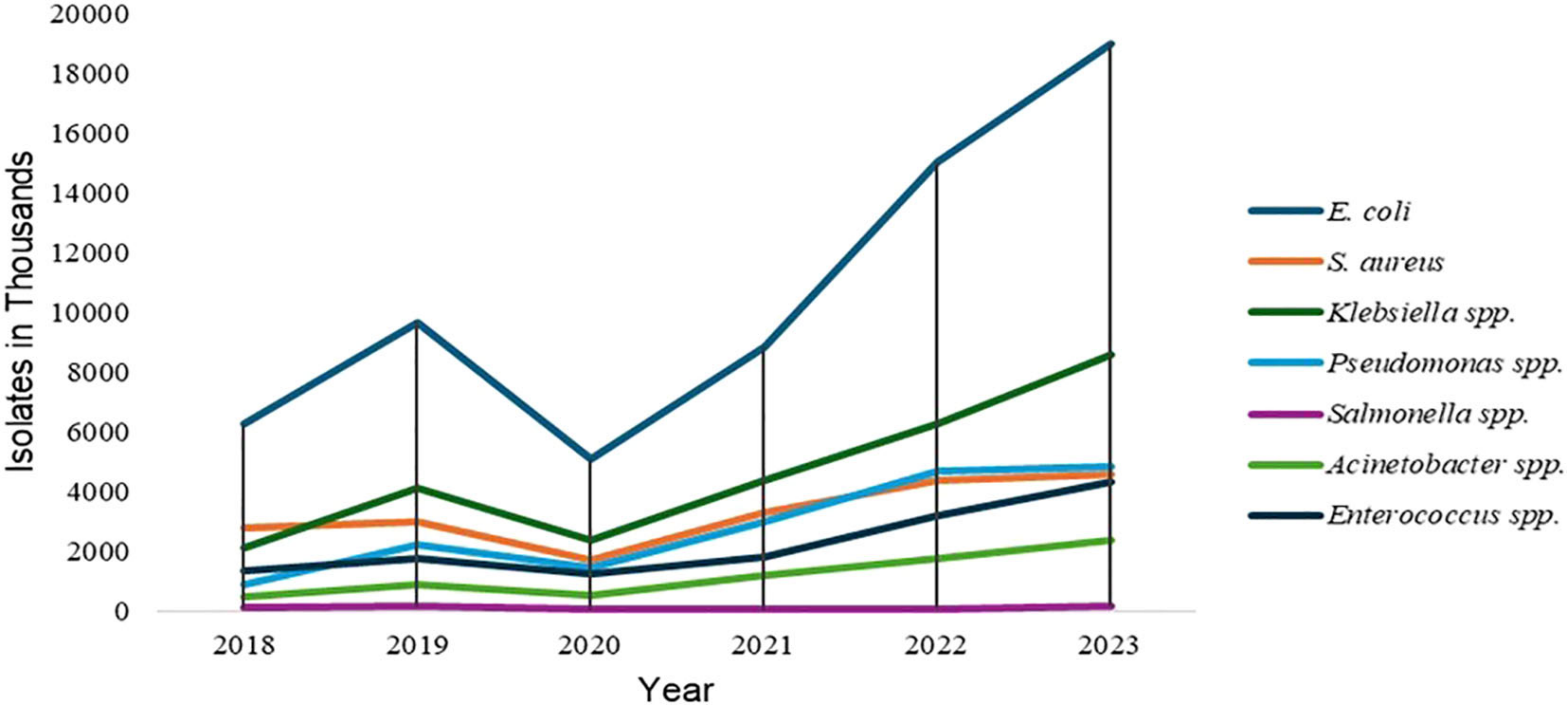
Year-wise prevalence trends of priority pathogens in OPD settings, adapted from NARS-Net annual reports 2018-23.

Antimicrobial resistance (AMR) refers to the ability of microorganisms, including bacteria, viruses, fungi and parasites, to resist the therapeutic effects of previously effective antimicrobials (Tang et al., 2023). By 2050, this silent pandemic could cause 10 million mortalities globally, up from 700,00 per year (Ahmed et al., 2024c). High prevalence of AMR in India stems from the extensive use of antibiotics in clinical settings (Gandra et al., 2017). Antimicrobial – resistant pathogens such as Carbapenem-resistant *Enterobacterales* (CRE), Multi-drug-resistant Gram-negative bacteria (MDR-GNB) such as *Acinetobacter baumanii*, *Pseudomonas aeruginosa* and Gram-positive cocci (GPCs) such as MDR *Staphylococcus aureus* whose management have become complicated, contribute significantly to hospital-acquired infections (HAIs). In (**Figure 1**) we have provided a graphical representation of current trends in AMR. These infections result in treatment failures, extended length of stays, increased healthcare expenses, and socioeconomic burden, for healthcare facilities (Jean et al., 2022; Kumar et al., 2021). In addition to Hospital acquired infections (HAIs), AMR is a growing problem in outpatient settings, where the prevalence of drug-resistant bacteria in community-acquired infections continues to rise (Alsan et al., 2018). Alarming rise in prevalence of resistant pathogens in OPD settings can be inferred from (**Table 1**; **Figure 2**). The WHO published its 2024 priority bacterial pathogens list, which classifies *Acinetobacter baumannii*, Carbapenem and 3^rd^ generation Cephalosporin resistant *Enterobacterales* as critical priority pathogens, while *Staphylococcus aureus, Pseudomonas, Enterococcus faecium, Shigella* spp., and *Salmonella* spp. as high priority pathogens. It is important to note that these pathogens have consistently been ranked highly antimicrobial-resistant ever since the first WHO report in 2017 (Jesudason, 2024; World Health, 2024a).

**Table 1:** Priority pathogens isolated and reported in OPD Settings; Data extracted from NARS – Net Annual Reports 2018 – 23.

Pathogens	2018	2019	2020	2021	2022	2023
*E. coli*	6,525	9,668	5,110	8,848	15,034	18,995
*S. aureus*	2,770	2,964	1,678	3,274	4,392	4573
*Klebsiella spp.*	2111	4,090	2,368	4,382	6,289	8,579
*Pseudomonas spp.*	894	2,202	1,422	2985	4700	4812
*Salmonella spp.*	102	150	46	35	83	150
*Acinetobacter spp.*	473	869	520	1,202	1,729	2,392
*Enterococcus spp.*	1,362	1,764	1,229	1,827	3,184	4,315

Several factors contribute to increase in AMR in India which includes: various secondary hospitals’ poor antimicrobial stewardship program due to poor resource availability, the absence of educational programs to fill the knowledge gap of healthcare professionals (Mathew et al., 2020), over-the-counter (OTC) dispensing of antibiotics, and patients refilling antibiotics using older prescriptions (Jani et al., 2021). In addition, an important factor in the emergence and spread of AMR is the improper use of antibiotics and lack of monitoring. To address this growing issue, the Indian Council of Medical Research (ICMR) developed evidence-based treatment guidelines. The updated information on treatment guidelines together with the dosage, mode of administration, and length of therapy for skin infections, soft tissues, bones, and joints, are included in the second edition. To provide uniformity in the management of different infectious diseases, the “Treatment guidelines for antimicrobial use in common syndromes” seek to justify the use of antibiotics (Aggarwal et al., 2019). Before starting antimicrobial therapy, antimicrobial susceptibility testing must be carried out extensively, especially to estimate minimum inhibitory concentration (MIC). Together with personalized medicine approaches, such as pharmacogenomics, that is treatment based on genetic profiling of patients and therapeutic drug monitoring of antimicrobial drugs, should be included. The use of narrow-spectrum antibiotics provides a targeted antimicrobial effect against specific pathogens, thus limiting the development of antimicrobial resistance. This approach ensures that antibiotics are administered precisely, within the minimum inhibitory concentration (MIC) levels, achieving optimal therapeutic efficacy. It simultaneously prevents drug-related adverse effects, including drug-induced liver injuries (DILIs), nephrotoxicity, ulcers, rashes, vomiting, and gastric upset (Roberts et al., 2012). If appropriate solutions are not implemented, AMR could compromise the sustainable development goals (SDGs) and cause millions of people to continue living in poverty by 2030 (World Health, 2021). Strong measures to prevent the spread are crucial, as evidenced by the prevalence of resistant pathogens in India (Ravishankar et al., 2021).

A recent systematic review on Knowledge, Attitudes and Practices (KAP) related to AMR reported that knowledge levels were highest among doctors, followed by nurses and pharmacists. However, despite relatively good theoretical knowledge, a noticeable gap persists in the translation of this knowledge into appropriate clinical practice among healthcare providers in India. Moreover, the review highlighted that the majority of published literature predominantly focuses on hospital-based infections, indicating a clear gap in evidence related to AMR in outpatient and community settings (Rana et al., 2024).

This review provides a unique perspective by integrating antimicrobial resistance (AMR) data from Indian healthcare, with a particular emphasis on outpatient settings that are often overlooked in existing literature. Unlike previous reviews that primarily focus on hospital-acquired infections, this article explores the community dimension of AMR and its implications for public health. Furthermore, it introduces an innovative framework linking AMR mitigation strategies with precision medicine approaches, including pharmacogenomics, metabolomics, proteomics, and transcriptomics. By combining multi-omics insights with national surveillance data and stewardship initiatives, this review highlights the translational potential of personalized antimicrobial therapy tailored to the Indian healthcare ecosystem. This integrated perspective offers a novel direction for AMR research and policy, bridging the gap between genomic science and clinical application in resource-limited settings.

## 2 Prevalence and AMR patterns of infectious diseases

### 2.1 Urinary tract infections

A wide range of bacterial genera that cause urinary tract infections (UTIs) include: *Escherichia, Klebsiella, Proteus, Pseudomonas, Enterococcus, Staphylococcus* sp*ecies, Enterobacter* sp*ecies*, and *Citrobacter* species (Ronald, 2002). The most prevalent uropathogens are GNBs, amongst which *E. coli* is the most reported cause of UTI and is followed by *Proteus* spp.*, Klebsiella* spp., and *Pseudomonas* spp. Whereas, around a quarter of infections are caused by gram-positive bacterium such as *Enterococcus* spp (Smita et al., 2019). A breakdown of prevalent bacterial genera in different types of urinary tract infections can be found in (**Table 2**) (Patel et al., 2021; Prabhu et al; Umesha et al., 2018; M et al., 2017; Srivastava et al., 2014).

**Table 2:** Pathogen composition of different types of Urinary Tract Infections.

Type of UTI	Prevalent Bacterial Pathogens
Acute Cystitis	*E. coli*
Acute Pyelonephritis	*E. coli, K. pneumoniae, Pseudomonas spp., Enterococcus spp., Proteus mirabilis, Citrobacter spp.*
Urosepsis	*E. coli, K. pneumoniae, E. cloacae, E. faecalis, E. faecium*, MRSA, MSSA
CA – UTI	*E. coli, P. aeruginosa, E. faecium, K. pneumoniae*, MRSA, *Enterobacter spp., Candida spp.*

Reportedly, MDR strains are highly prevalent in India (Mohapatra et al., 2022). Significant resistance to beta-lactam antibiotics, beta-lactamase inhibitor combinations such as piperacillin-tazobactam, third-generation cephalosporins, fluoroquinolones (e.g., ciprofloxacin), and an increasingly concerning rise in resistance to carbapenems which are usually saved for severe infections have been observed in *E. coli.* While *Klebsiella* and *Proteus* species have comparable patterns of resistance demonstrating strong resistance to third-generation cephalosporins, fluoroquinolones, and nitrofurans. High levels of resistance to beta-lactams, cephalosporins, fluoroquinolones, and aminoglycosides are seen in *Pseudomonas* spp., a challenging pathogen to treat. Beta-lactams, nitrofurans, aminoglycosides, tetracyclines, and some incidences of vancomycin resistance are among the antibiotics against which *Enterococcus* spp., exhibit alarming patterns of resistance (Smita et al., 2019; Mohapatra et al., 2022).

### 2.2 Respiratory tract infections

Airborne pathogens spread quickly, especially in crowded, poorly ventilated situations. Respiratory infections are the most prevalent infectious diseases in the world. From minor ailments like the common cold to potentially fatal ones like pneumonia, they can range in severity. These illnesses fall under the category of upper respiratory tract infections (URTIs), which impact the sinuses, pharynx, larynx, and nose. Airway and lung complications comprise lower respiratory tract infections (LRTIs). It is relatively rare for URTIs to have bacterial origins; instead, viruses, such as rhinoviruses, are usually the source (Jain et al., 2001). Common bacterial respiratory infections include pneumonia, bronchitis, community acquired pneumonia (CAP). Common causal bacterial genera for such diseases include *Streptococcus pneumoniae, Staphylococcus* spp.*, Mycoplasma* spp.*, Haemophilus influenzae, Klebsiella pneumoniae*, and *Pseudomonas* spp (Feldman and Shaddock, 2019).

Studies indicate GNB were the most common causes of infections, amongst which *Pseudomonas aeruginosa*, and *Klebsiella pneumoniae* were the most isolated organisms (Singh et al., 2020). Additionally, gram - positive bacteria *Streptococcus pneumoniae* is the leading cause of pneumonia in hospitalized patients (Sangale et al., 2021).

MDR *Klebsiella pneumoniae* and *Pseudomonas aeruginosa* strains show very high levels of resistance towards cephalosporins (ceftriaxone), fluoroquinolones (ciprofloxacin), beta lactam/beta lactamase inhibitors (piperacillin -tazobactam) have been reported (Kaleem Ullah et al., 2022; Sharma et al., 2023b). *Streptococcus pneumoniae* showed high levels of resistance for macrolides (Erythromycin), an increase in resistance for fluoroquinolones (Levofloxacin), and a concerning increase in resistance towards carbapenems (Meropenem) (Kulkarni et al., 2023).

### 2.3 Skin and soft tissue infection

Microorganisms that give rise to SSTIs are generally divided into transitory pollutants and resident skin flora. While resident bacteria on the skin typically coexist harmlessly, they might turn infectious in rare situations such as trauma or immunosuppression. *Staphylococcus aureus, Staphylococcus warneri, Streptococcus pyogenes*, and *Corynebacterium* species are typical transitory flora. Additionally, GNB are often implicated, including *Proteus* species, *Escherichia coli*, and *Pseudomonas aeruginosa* (Tognetti et al., 2012).

In India, studies show a predominance of Gram-positive cocci (GPC) in SSTIs in comparison to GNB (Roopashree et al., 2021). Amongst GPCs, *S. aureus* is the most prevalent, with studies pointing towards an increase in prevalence of Methicillin-resistant *S. aureus* (MRSA) strains. Many *S. aureus* isolates showed high levels of resistance towards penicillin and cephalosporin drugs (Roopashree et al., 2021; Shah et al., 2022). MDR *P. aeruginosa* and *E. coli* were the most prevalent GNBs, being highly resistant to penicillin and penicillin derivative drugs, cephalosporins, beta lactams/beta-lactamase inhibitors (Deka et al., 2020; Kakhandki et al., 2020; Ramakrishna et al., 2021).

### 2.4 Diarrhoeal infections

Diarrhoea can be caused by different infectious agents such as bacteria (*E. coli, Salmonella, Shigella, Vibrio*), viruses (*Rotavirus, Adenovirus*), and protozoans (*Giardia, Cyclospora, Cryptosporidium, Isospora, Microsporidia*) (Shrivastava et al., 2017).

Diarrheagenic *E. coli* (DEC) strains are most prevalent and show resistance towards beta-lactams (Ampicillin), macrolides (Azithromycin), fluoroquinolones (Ofloxacin, Ciprofloxacin), and tetracyclines (Doxycycline) (Biswas et al., 2024). *Shigella* sp*ecies* were also frequently seen, with *S. flexneri* being the most prevalent, followed by *S. sonnei, S. dysentriae* and *S. boydii*. *Shigella* spp., showed high resistance towards fluoroquinolones (Ofloxacin, Ciprofloxacin, Nalidixic acid, Norfloxacin), tetracyclines (Tetracycline), sulfonamides (Co-trimoxazole), aminoglycosides (Streptomycin) and a growing resistance towards amphenicols (chloramphenicol) and beta lactams (Ampicillin) (Bose et al., 2024; Jain et al., 2020).

Amongst *Salmonella* spp.*, S. typhi*, the most common species showed high resistance towards fluoroquinolones (Ciprofloxacin, Nalidixic acid) and increasing resistance towards cephalosporins (Cefixime, Ceftriaxone) (Biswas et al., 2022). Nontyphoidal *Salmonella* (NTS) also were moderately prevalent, showing MDR patterns encompassing fluoroquinolones (Ciprofloxacin, Levofloxacin), aminoglycosides (Gentamycin), macrolides (Azithromycin), and sulphonamides (Trimethoprim/Sulfamethoxazole) (Mahindroo et al., 2024).

### 2.5 Hospital acquired infections

In India, GNBs such as *P. aeruginosa, Klebsiella* spp.*, Acinetobacter baumanii, Citrobacter* spp.*, Aeromonas* spp., are the most prevalent organisms causing ventilator associated pneumonia (VAP). Among GPC, *Staphylococcus aureus* is also a significant contributor to VAP cases. *Enterococcus* spp. showed very high prevalence in catheter-associated urinary tract infections (CAUTI) followed by *Klebsiella* spp.*, Citrobacter* spp., showing significant prevalence. For catheter-related bloodstream infections (CRBSI), the leading pathogens are *P. aeruginosa, Klebsiella* spp.*, Acinetobacter baumanii, Citrobacter* spp., and *S. aureus* (Kumar et al., 2021; Saikeerthana et al., 2021; Dr. Ranjeet et al., 2024). (**Table 3**) (Crank and O’Driscoll, 2015; Hassoun et al., 2017; Nandhini et al., 2022; Singhal et al., 2022) provides insights of treatment options for hard-to-treat bacterial diseases.

**Table 3:** Associated diseases and treatments for Methicillin-Resistant Staphylococcus Aureus (MRSA) and, Vancomycin-Resistant Enterococcus (VRE) infections.

Pathogen	Associated Diseases	Treatment options
MRSA	Uncomplicated and Complicated Bacteraemia, Infective endocarditis, Skin and soft tissue infections, Sepsis, etc	Vancomycin and Teicoplanin are choice antibiotics. Other antibiotics include ceftraoline, daptomycin, oxazolidones and Linezolid
VRE	Bacteraemia, Infective endocarditis, Intra-abdominal and pelvic infections, Urinary tract infections, Skin infections, etc	Linezolid, Tedizolid, Quinupristin or Dalfopristin, Daptomycin, Tigecycline, Telavancin, Dalbavancin, Oritavancin

A concerning trend in Indian healthcare is the alarming rate of AMR among hospitalized patients in India. A significant number of bacterial isolates have been classified as MDR, and an even greater proportion are suspected to be extremely-drug resistant (XDR). Particularly, many strains of *P. aeruginosa, Acinetobacter baumanii* and *Klebsiella* spp., have shown XDR patterns. Furthermore, many isolates of *S. aureus* have also been reported as MDR (Vaithiyam et al., 2020). An alarming increase in carbapenem - resistant *Enterobacteriaceae* (CRE) is reported amongst which *Klebsiella* spp. is the most reported CRE showing high levels of resistance towards carbapenems (Modi et al., 2021).

## 3 Factors contributing to AMR in India

### 3.1 Antibiotic misuse and overuse in India

Global estimates indicate that only 50% of the global antibiotic consumption is appropriately justified (Iskandar et al., 2020). Dispensing antimicrobial drugs without prescription by pharmacies in the private sector in India within an urban setting was unacceptably high (around 67%) (Shet et al., 2015). Reports indicate that 44% of outpatient antibiotic prescriptions are intended to inappropriately treat acute respiratory conditions (e.g., viral upper respiratory tract infections, bronchitis, asthma, allergies, and influenza) (Laxminarayan and Chaudhury, 2016). In India nearly 60% mortality of children aged 5 to 14 years is due to infectious diseases such as diarrhea, pneumonia, measles, etc (Morris et al., 2011). The rate of infectious disease is also linked with increased sale and consumption of antibiotic in India. The lack of diagnostic facilities, irrational prescription, lack of knowledge, experience about antibiotic use and pharma companies’ incentives are the factors which cause misuse of antibiotics.

### 3.2 Unregulated OTC dispensing of antibiotics

A 2018 study found that more than 60% of fixed drug combinations (FDCs) had no regulatory approval (McGettigan et al., 2019). They found many formulations were pharmacologically incompatible, reinforcing the need for better regulation and oversight of the pharmaceutical industry. Schedule H1, an amendment to the Drugs and Cosmetics Rules Act of 1945 that imposed restrictions on OTC dispensing of certain antibiotics (mostly third and fourth-generation cephalosporins, carbapenems, and newer fluoroquinolones) was perceived as a vital policy with ineffective implementation and adherence (Nair et al., 2023). Universal coverage of vaccines is expected to prevent 11.4 million days of antibiotic use per year in children less than 5 years of age. Consequently shows an estimated 47% reduction in antibiotics used to treat these pneumococcal infections (Wu et al., 2021).

### 3.3 Economic and healthcare access barriers contributing to AMR

Due to healthcare costs, patients avoid diagnostic and purchase antibiotics without a prescription and self-medicate with older prescriptions to have a rapid recovery. These collectively promote the misuse of antibiotics and increase AMR (Jani et al., 2021). In rural areas, many healthcare providers are often inefficient and employ unqualified staff. Thereby, urging the patients to choose private healthcare, which leads to high costs, particularly for the middle-class and low-income earners. To cut this expense, patients turn to self-medication, which results in high consumption of antibiotics. The observation validates the finding of Wu et al (Wu et al., 2021), which highlighted a rise in parents self-medicating their children (below 5 yrs), posing a serious threat to child health. This behavior causes AMR to first-generation and broad-spectrum antibiotics (Gandra et al., 2017).

### 3.4 Environmental contribution to AMR

Antibiotics are extensively used to compensate for poor sanitation, prevent diseases in herds/flocks, treat bacterial infections, and to promote animal growth in the distinct agricultural sectors (Page and Gautier, 2012). The magnitude and contribution of cross-reservoir resistance transmission from animals to humans is difficult to quantify and track mainly because of globalized food trade (Mather et al., 2013). Wastewater is flagged as a significant environmental reservoir of drug-resistant bacteria; horizontal gene transfer of antimicrobial resistance genes (ARGs) results in the dissemination of AMR in microbial communities. India has become the center for MDR and XDR *Mycobacterium tuberculosis*, which complicates the control of tuberculosis. In addition, the genomic flexibility of many resistant organisms allows them to acquire resistance to reserved antibiotics like carbapenem and colistin (Jani et al., 2021).

## 4 Impact of AMR on clinical outcomes

### 4.1 Effect of AMR on patient morbidity and mortality

People’s health is directly impacted by antibiotic resistance. It impairs our capacity to create a successful treatment strategy to eradicate infectious diseases and lower rates of morbidity and death. Patients who become resistant to many drugs are more likely to experience higher morbidity or even mortality (Cornejo-Juárez et al., 2015). AMR poses a risk to the treatment of pre-operative and post-operative nosocomial infections, making treatments difficult for already debilitated patients. The burden of patient mortality is as high as 13.1% overall in MDR infections, with MDR *A. baumanii* infections resulting in 2–3 fold higher mortality (29%) (Barrasa-Villar et al., 2017; Massart et al., 2022; Figueiredo Costa, 2008). However, it to be noted that long course colistin treatment over short course for Carbapenem resistant *Acinetobacter baumanii* proved to be promising and beneficial for critically ill patients and helped in reduction of 30-day mortality rate (aOR = 0.46, 95% CI: 0.26 – 0.83, p = 0.009) amongst the long course colistin group with negligible differences in nephrotoxicity amongst the two groups (aOR 1.28, 95% CI: 0.74 – 2.22, p = 0.368) (Katip et al., 2023).

For hospitalized patients, increased length of ICU stay is higher in patients with HAIs, averaging at 13 days, and is also associated with a higher risk of mortality (Ramsamy et al., 2017). Similarly, for community-acquired infections, MDR infections were associated with inappropriate antibiotic use and were directly related to higher mortality (adjusted odds ratio (aOR), 6.06, 95% CI, 1.2 – 55.7) (Lohiya et al., 2020). Bacteraemia, being a life-threatening condition, often resulting in half of the patients succumbing to illness, requires exhaustive antimicrobial treatment. Due to ever-increasing AMR, antimicrobial therapy is often ineffective resulting in increased patient mortality (Figueiredo Costa, 2008; Ramsamy et al., 2017). Additionally, it makes the treatment plan more difficult for patients with long-term antibiotic-dependent conditions such as tuberculosis, whose prevalence is already rampant in India (Lohiya et al., 2020).

### 4.2 Rural AMR gaps

Antimicrobial resistance (AMR) has become a widespread public health concern across India; however, significant differences in resistance patterns and underlying drivers are observed between rural and urban settings. These variations are influenced by multiple socio-economic, environmental, and healthcare-related factors. Urban areas, in particular, are often densely populated and experience higher levels of environmental pollution, conditions that facilitate the rapid transmission of infectious diseases and potentially accelerate the emergence and spread of AMR (Balachandra et al., 2021).

In contrast, rural areas may have comparatively lower access to antibiotics due to limited healthcare infrastructure; however, inappropriate antibiotic use remains a significant concern. Limited awareness regarding proper antibiotic usage, coupled with widespread over-the-counter (OTC) availability, contributes to misuse in rural India (Sharma et al., 2023a). The situation is particularly alarming in states such as Bihar—India’s third most populous state—where scarce healthcare resources and inadequate infrastructure exacerbate the problem. Evidence indicates a substantial rise in antimicrobial resistance to β-lactams, carbapenems, and fluoroquinolones in Bihar. Methicillin-resistant Staphylococcus aureus (MRSA) prevalence is notably high at 65%, significantly exceeding the national average of 47.8% (Modgil et al., 2025). Furthermore, community-level comparative studies between urban and rural settings demonstrate a consistently higher prevalence of resistant pathogens in rural regions, with rural AMR trends surpassing corresponding data reported by ICMR.

### 4.3 Economic impact of AMR on healthcare

It is now evident that MDR infections take a significant toll on patient and hospital finances. Studies pointing towards increased length-of-stay (LOS) and increased patient mortality can be extrapolated to higher expenditures on patient health (Nathwani et al., 2014). AMR increases the cost of healthcare due to additional nursing and medical care needed for the patient (Pulingam et al., 2022). Treatment of drug-resistant pathogens inadvertently promotes further utilization of antimicrobials to curb the infection. This never-ending cycle results in the burden of purchasing costly antibiotics (Dadgostar, 2019). The median total cost of managing resistant infections in India is calculated at US$ 199 in comparison to US$ 109 for susceptible infections, the median cost of hospitalization per day is US$ 65 and US$ 35 for resistant and susceptible infections respectively. This resulted in heavy economic burdens as nearly half of the patients borrowed money for treatment expenses (Kadam et al., 2024).

### 4.4 Global significance

Outpatient AMR patterns in India provide valuable insights for healthcare professionals worldwide. India’s high antibiotic use and diverse resistance profiles mirror challenges faced by many low- and middle-income countries, offering a model for strengthening global surveillance and stewardship. Data from India support WHO-led initiatives such as GLASS, enhancing evidence-based AMR monitoring and policy development (World Health, 2014; Okeke et al., 2024). The AMR patterns observed in India can help healthcare professionals globally understand prescription trends, antibiotic guideline, and AMSP practice-related resistance mechanisms. Beyond national policy reforms, these insights can inform global antibiotic use strategies and guide the evolution of international frameworks for rational and precision-based antimicrobial therapy.

## 5 Current efforts and interventions to curb AMR

### 5.1 National AMR surveillance programs in India

**Figure 3:**
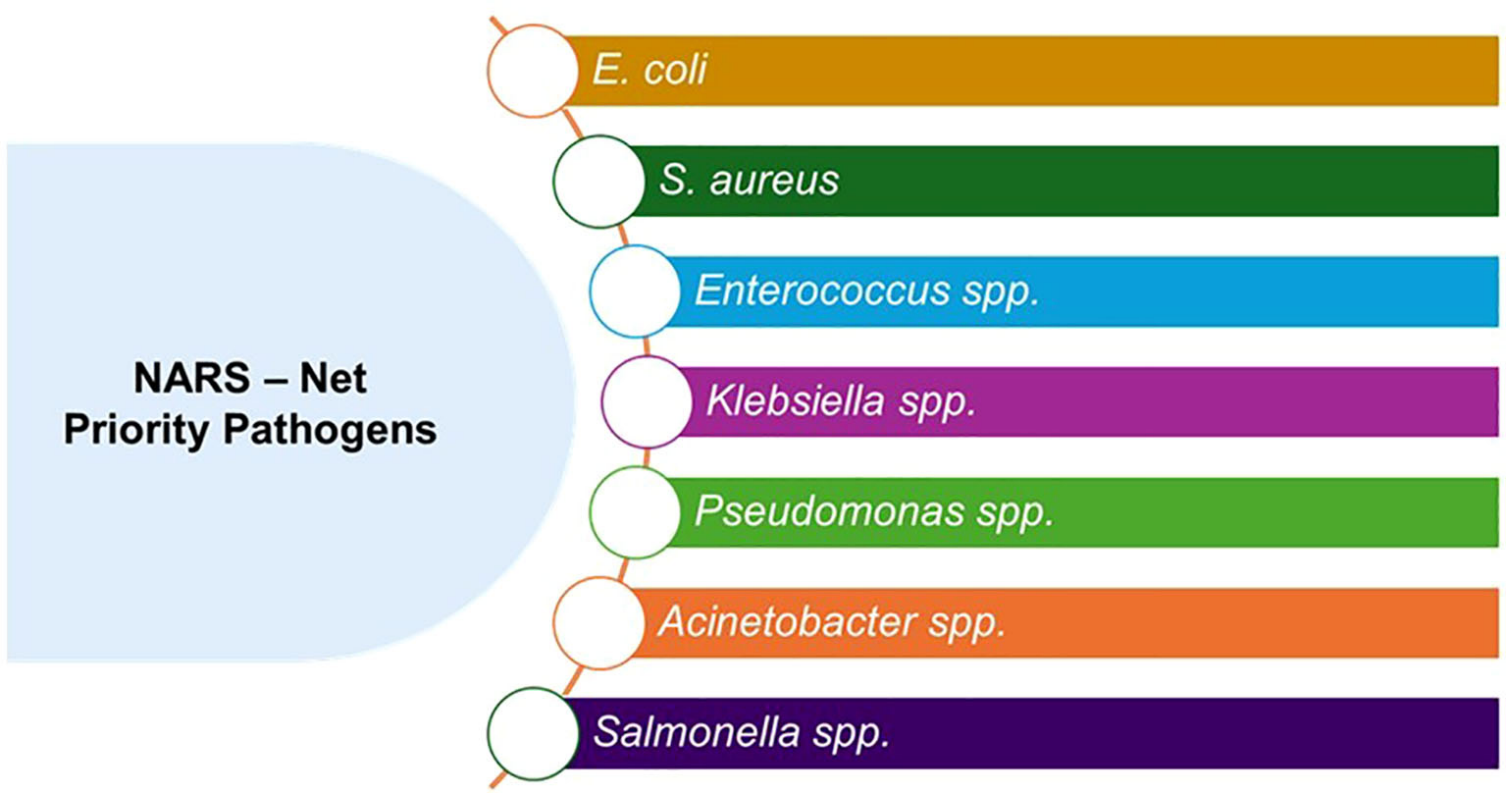
List of priority pathogens included under national antimicrobial resistance surveillance network (NARS-Net India).

According to the Global Burden of Disease study, bacterial infections cause around 37 million deaths globally, making AMR among dangerous bacteria a significant health problem (Murray et al., 2022). The 68^th^ World Health Assembly created and accepted the Global Action Plan on AMR (GAP–AMR) in 2015 after receiving support from several international organizations (World Health, 2024b). The AMR Political Declaration adopted by the 71^st^ UN General Assembly in 2016 affirmed the GAP-AMR and its five strategic objectives as the blueprint for addressing AMR globally and has informed the development of National Action Plans (NAPs). By the end of 2023, 178 countries had developed NAPs based on this framework with 68% implementing their plans, 25% having costed and budgeted NAPs and with Monitoring & Evaluation frameworks. However, so far only 10% of countries have responded that they have made specific financial provisions for implementing their NAPs which highlights the cross-cutting nature of AMR interventions.) (Gupta and Bhatia, 2023). In India, the 12th (2012-2017) five-year plan included the National Program on AMR Containment, which was started in 2013 and is managed by the National Centre for Disease Control (NCDC). The National Antimicrobial Resistance Surveillance Network (NARS-Net) was gradually extended to all states and Union Territories (UTs). (**Figure 3**) illustrates priority pathogens monitored under NARS - Net. Because of this effort, by March 2024, the program had 50 medical colleges and labs in 27 states and 6 UTs, guaranteeing geographic representation. After certification, “WHONET” an offline, open-source program for managing microbiological data, is used by the NCDC to evaluate surveillance data from NARS-Net sentinel sites. The 5-year NAP-AMR (2017–2021) was a crucial foundation for states to create action plans to address AMR locally because India’s healthcare system is state-governed (Nair et al., 2021). Similarly, ICMR created an AMR monitoring network (AMRSN) to gather nationally representative data on the trends and patterns of antibiotic resistance in pathogens of public health significance.

### 5.2 Precision medicine

Current treatment practices can be boiled down to a “one-size-fits-all” approach (Merker et al., 2020). Conventional antibiotic therapy comprises either a single dose or a combination of two or more antibiotics. Limitations of this approach relate to issues such as misuse, overuse and underuse of antibiotics. These increased risk of adverse effects and toxicities, also reducing the efficacy of antibiotics (Ahmed et al., 2024a; Wawruch et al., 2002).

Precision Medicine helps to make customized and informed medical decisions, tailoring therapies by considering patients’ genetic makeup, immune response, specific infection, and socio-environmental and lifestyle factors. Precision medicine affirms the belief that every patient has different demands rather than a “one-size-fits-all” approach (Naithani et al., 2021). Such informed therapies allow for better treatment outcomes by selecting the most appropriate antibiotics, quick modifications of antibiotic regimens, and allowing for better antimicrobial stewardship. These ultimately prevent the development of AMR (Ahmed et al., 2024a; Watkins, 2022).

#### 5.2.1 Pharmacogenomics

Precision medicine’s patient-centric approach allows us to closely monitor drug levels using therapeutic drug monitoring (TDM). This helps maintain the peak therapeutic efficacy of drugs while mitigating adverse effects and drug-related toxicities (Cusumano et al., 2020). Incorporating pharmacogenomic testing for single nucleotide polymorphisms (SNPs) and mutations brings out insights about patients’ responses towards the antimicrobial drugs prescribed to them. Thereby preventing unnecessary adverse drug reactions (ADRs) and toxicities promoting safe, effective, and conserved use of antimicrobial drugs (Daly, 2023). (**Table 4**) gives a detailed breakdown of genes, their Single nucleotide polymorphisms (SNPs) and the associated negatory effects (Hahn et al., 2016; Cirulli et al., 2019; Naidoo et al., 2017, 2018; Weiner et al., 2007, 2018; Andrews et al., 2010; Yee et al., 2013; Allegra et al., 2018a; Franke et al., 2011; Lancaster et al., 2012; Baietto et al., 2015; Allegra et al., 2018b; Chen et al., 2024; Estivill et al., 1998; Prezant et al., 1993; Gervasoni et al., 2013; Wang et al., 2012). SNPs that are related to various ADRs in persons carrying those mutations. These adverse effects include drug induced liver injuries (DILI), drug reactions with systemic eosinophilia (DRESS), nephrotoxicity, ototoxicity resulting in hearing loss, neutropenia, neurological side effects and immunologic adverse reactions (such as cutaneous adverse reactions due to polymorphisms in MHC Class I and II) a detailed breakdown is given in (**Table 5**) (Lucena et al., 2011; Stephens et al., 2013; Urban et al., 2017; Konvinse et al., 2019; Yang et al., 2017). An in-depth review of the pharmacogenomics of antibiotics (Stocco et al., 2020). Pharmacogenomic interpretation and screening for such polymorphisms will be of immense clinical importance; further research to prove such SNPs as biomarkers are required.

**Table 4:** Pharmacogenomic variants influencing antibiotic response and toxicities.

Antibiotic	Gene	rs no of gene	Effects	References
Beta lactams	ABCC4	rs1751034	Neutropenia	(Hahn et al., 2016)
Amoxicillin clavulanate (Penicillins)	PTPN22	rs2476601	DILI	(Cirulli et al., 2019)
Moxifloxacin	UGT1A, ABCB1, SLCO1B1	rs8175347, s3755319, rs2032582, s1045642, rs4149015	Altered drug clearance, reduced bioavailability, Altered absorption, increased blood drug levels	(Naidoo et al., 2017, Naidoo et al., 2018, Weiner et al., 2007, Weiner et al., 2018)
Flucoxacillin	PXR	rs3814055	Flucoxacillin induced DILI	(Andrews et al., 2010)
Cefotaxime (Cephalosporin)	SLC22A8	rs11568482	Lower renal clearance	(Yee et al., 2013)
Ceftriaxone (Cephalosporin)	ABCC2, ABCG2	rs2273697, rs13120400	Higher drug concentration in CSF	(Allegra et al., 2018a)
Erythromycin (Macrolide)	ABCC2 SLCO1B1	rs717620 rs4149056	Increased drug clearance Reduced drug clearance	(Franke et al., 2011, Lancaster et al., 2012)
Daptomycin	ABCB1	rs1045642	Decreased drug clearance	(Baietto et al., 2015)
Linezolid	ABCB1	rs1045642	Lower clearance	(Allegra et al., 2018b)
Aminoglycosides	Mitochondrial 12S rRNA	rs267606617	Irreversible bilateral ototoxicity, reversible nephrotoxicity	(Chen et al., 2024, Estivill et al., 1998, Prezant et al., 1993)
Levofloxacin	ABCB1, ABCG2	rs2032582, rs1045642 rs2231142	Seizures, neurological side effects.	(Gervasoni et al., 2013)
Trimethoprim-sulfamethoxazole	GCLC	rs76114	DRESS	(Wang et al., 2012)

ATP-Binding Cassette Subfamily C Member 4 (ABCC4), Protein Tyrosine Phosphatase, Non-Receptor Type 22 (PTPN22), UDP glucuronosylytransferase family 1 (UGT1A), ATP-Binding Cassette Subfamily B Member 1 (ABCB1), Solute Carrier Organic Anion Transporter Family Member 1B1 (SLCO1B1), Pregnane X Receptor (PXR), Solute Carrier Family 22 Member 8 (SLC22A8), ATP-Binding Cassette Subfamily C Member 2 (ABCC2), ATP-Binding Cassette Subfamily G Member 2 (ABCG2), Solute Carrier Organic Anion Transporter Family Member 1B1 (SLCO1B1), ATP-Binding Cassette Subfamily B Member 1 (ABCB1), ATP-Binding Cassette Subfamily G Member 2 (ABCG2), Glutamate-Cysteine Ligase Catalytic Subunit (GCLC)

**Table 5:** Antibiotics’ adverse drug reactions association with MHC Class I and II polymorphisms; DILI- Drug Induced Liver Injury.

Antibiotics	SNPs / Polymorphisms	Adverse drug reactions	References
Amoxicillin clavulanate (Penicillins)	HLA – A*30:02, HLA – B*18:01	DILI	(Lucena et al., 2011; Stephens et al., 2013)
Minocycline	HLA – B*35:02	DILI	(Urban et al., 2017)
Vancomycin	HLA-A*32:01	DRESS	(Konvinse et al., 2019)
Clindamycin	HLA – B*51:01	Cutaneous adverse reactions	(Yang et al., 2017)

#### 5.2.2 Metabolomics

Carbapenem-resistant *Acinetobacter baumannii* (CRAB) strains are prevalent worldwide and a significant threat to public health. Orthogonal Partial Least- Squares discriminant analysis (OPLS-DA) identified key biomarkers distinguishing Carbapenem-resistant *Acinetobacter baumannii* (CRAB) and Carbapenem-sensitive *A. baumannii* (CSAB). A total of 16 differential metabolites, including AMP, guanine, glutamine, fumarate, succinic acid, and α-ketoglutarate, were detected. Among these, eight metabolites were downregulated in CRAB, with six associated with the tricarboxylic acid (TCA) cycle and purine metabolism pathways (Li et al., 2024). An interesting example of discovering a metabolite-based antibiotic adjuvant by comparing the metabolic profiles of Gentamicin-sensitive *Salmonella Choleraesuis* (SCH-S) and gentamicin-resistant *Salmonella Choleraesuis* (SCH-R). Remarkably, D-ribose was the most suppressed metabolite in SCH-R and enhanced the efficacy of gentamicin against SCH-R and clinical multidrug-resistant strains (Zhou et al., 2022). Glucose and alanine are prominent biomarkers whose abundances are significantly reduced in kanamycin-resistant *Edwardsiella tarda* (Peng et al., 2015).

The potential mechanisms of utilizing gas chromatography-mass spectrometry (GC-MS) based metabolomics approaches are explored to profile the metabolomes of *Edwardsiella tarda* in the presence or absence of serum stress. They identified exogenous succinate as promoting the TCA cycle and increasing serum resistance, while TCA cycle inhibitors (bromopyruvate and propanedioic acid) that inhibit the TCA cycle attenuated serum resistance (Cheng et al., 2017). The metabolic profiles of clinically isolated multidrug-resistant and susceptible *Escherichia coli*, identifying glutamine as a potential biomarker that is suppressed in drug-resistant strains (Zhao et al., 2021). The differential metabolomes of *S. agalactiae* evades serum-mediated killing. Through bioinformatics analysis, decreased malic acid and increased adenosine are identified as the most crucial biomarker. The findings reveal that *S. agalactiae* utilizes a metabolic trick to respond to plasma killing due to serum resistance (Wang et al., 2016). To determine resistance profiles of *A. baumannii* (ATCC strain), 26 clinical isolates were screened against ciprofloxacin, colistin, cefixime, gentamicin, and co-amoxiclav. Malleobactin, Endophenazine, pyochelin, and L-lysine were significantly associated with ciprofloxacin resistance while Cefixime resistance is associated with L-serine and cytidine (Ramzan et al., 2025). A total of 40 metabolites were differentiated between ciprofloxacin. Susceptible and resistant isolates of *Klebsiella oxytoca*. Univariate receiver operating characteristic (ROC) curve analyses revealed that six of these metabolites, glycerol-3-phosphate, O-phosphoethanolamine, asparagine dehydrate, maleimide, tyrosine, and alanine, have a crucial role in distinguishing susceptible from resistant isolates (AUC>0.84) contributing to antimicrobial resistance. The identified metabolites belong to central carbon metabolism, arginine and proline metabolism; alanine, aspartate, and glutamate metabolism; and pyruvate metabolism (Ahmed et al., 2024b).

#### 5.2.3 Proteomics

Whole-genome sequencing and proteome analysis of *Escherichia coli*, have decoded the dominant Antimicrobial Resistance Genes *bla_CTX-M-15_*, *bla_CMY-42_*, *bla*_NDM-5_, and *aadA* (Mat Ghani et al., 2025) and *bla*_TEM-1B_, *bla*_OXA-232_, *bla*_NDM-1_, *rmtB*, and *rmtC* were dominant in *Klebsiella pneumoniae*. In contrast, *Pseudomonas aeruginosa* and *Acinetobacter baumannii* harbored *bla_VEB_*, *bla_VIM-2_*, *aph(*3’), *strA/B*, *bla_OXA-23_*, *aph*(3′) variants, and *amrA*, respectively (Mehrotra et al., 2023). *Acinetobacter baumannii* strains revealed the role of *bla*_OXA-23_ and *bla*_OXA-66_ genes in carbapenem resistance. Through 16S rRNA gene sequence analysis and species-specific PCR targeting, the *bla*_OXA51_-like gene has been identified. Comparative genome analysis revealed *bla*_OXA-66_ as the most dominant variant of *bla*_OXA-51_-like gene and a widespread distribution of *bla*_OXA-23_ gene (Mat Ghani et al., 2025).

Multidrug-resistant *E. coli* proteome under antimicrobial pressure highlights a significant role for chromosomally encoded genes. Several proteins with altered abundance in response to ampicillin, cefotaxime, and imipenem, compared to untreated controls, have been identified. These include stress-related proteins such as ecotin (Eco) and methionine-R-sulfoxide reductase (MsrC) and detoxification proteins like SodA (ampicillin and cefotaxime), OsmC, GrxC, GrxD (cefotaxime), OxyR (imipenem), and PspE (cefotaxime and imipenem). Cold shock proteins (CspE, CspA) were elevated in cefotaxime, imipenem, and ampicillin-treated groups. Additionally, β-lactamase CTX-M-15 was significantly upregulated under cefotaxime exposure and TEM-1 under imipenem, but neither in response to ampicillin (Margalit et al., 2022). These findings partially differ from Møller et al. and can be attributed to differences in β-lactamase variants, antibiotic concentrations, and plasmid content (Møller et al., 2017). *Bacteroides fragilis* can be classified into division I (*cfiA* negative - chromosomal carbapenemase gene negative) and division II (*cfiA* positive- chromosomal carbapenemase gene positive) isolates. 14.9% proportion of *cfiA-*positive *B. fragilis* among blood culture isolates. Division II (*cfiA* positive) *B. fragilis* is more resistant to β-lactam antibiotics than division I (*cfiA* negative), making *cfiA* a valuable biomarker for antimicrobial therapy. Empiric treatment for Division II isolates should avoid carbapenems and co-amoxiclav (Jeverica et al., 2019).

An easy and rapid protocol for detecting KPC (*Klebsiella pneumoniae* carbapenemase) by MALDI-TOF MS is developed from the colony, and positive blood culture of *Klebsiella pneumoniae and Escherichia coli*. Statistical results showed 100% sensitivity, CI 95%: [94.0%; 100%] and 100% specificity, CI 95%: [94.6%; 100%], indicating a promising test with a high discriminative power. This approach offers a cost-effective and clinically impactful tool for early detection of KPC-2, supporting timely and targeted antibiotic stewardship in *Enterobacterales* infections (Figueroa-Espinosa et al., 2019). The “MALDIxin” test, a MALDI-TOF MS-based diagnostic that identifies polymyxin resistance in intact *Escherichia coli* within 15 minutes. The assay detects pETN-modified lipid A and distinguishes between chromosomal and plasmid-encoded resistance mechanisms. MALDIxin test accurately identified all *mcr-1* producers in a blinded panel of carbapenemase-producing *E. coli* (Dortet et al., 2018).

#### 5.2.3 Transcriptomics

There are various transcriptome biomarkers which actively promote *Pseudomonas aeruginosa* resistance to several drug classes. The *algX* gene, which stimulates alginate production, most likely enhances the protection for fluoroquinolones like ciprofloxacin by facilitating the formation of biofilms. In addition, the expression levels of the putative proteins like PA14_32290, PA14_48950, and PA14_59390 also indicated resistance against antibiotics. Furthermore, resistant strains showed an increased expression of the *opdP* gene, which codes for a glycine-glutamate dipeptide porin, and *yedZ* (PA14_62100), a sulfide oxidase subunit. One important factor in the development of multidrug resistance, particularly ciprofloxacin resistance, was the sensor kinase gene *cbrA* (Khaledi et al., 2016). The *ampC* gene showed a strong connection with ceftazidime resistance for β-lactam antibiotics such as ceftazidime and meropenem, with higher minimum inhibitory concentrations (MICs) correlating to greater expression levels (Libbrecht and Noble, 2015; Sundaramurthy and Eghbalnia, 2015).

The *oprD* gene, which codes for a carbapenem entry porin, downregulated in meropenem-resistant strains. The relevance of this downregulation in resistance was validated by protein studies and nonsense mutations, even though it did not reach statistical significance by AUC. In addition, the meropenem resistance pattern was influenced by the increased expression of genes such *gbuA* (a guanidinobutyrase involved in the arginine dehydrogenase pathway), cc4 (encoding the diheme protein cytochrome c4), and PA14_46110 (a putative sodium-solute symporter). When combining these indicators, it provides helpful targets for clinical testing and aid in the prediction of resistance phenotypes (Köhler et al., 1999).

### 5.3 Role of antimicrobial stewardship programs and infection control measures

AMR is a significant threat to public health, driven by several key issues. These include inappropriate and overuse of antibiotics in humans and agriculture, and poor infection control and management. This is further exacerbated by the inaccessibility of clean drinking water and proper sanitation, increased travel and globalization, and the slow development of novel or improved antimicrobial drugs (Mukherjee, 2024). The management of antimicrobial resistance now requires antimicrobial stewardship, which seeks to maximize antibiotic use through coordinated actions across healthcare settings, especially considering the slow development of new antibiotics (Ha et al., 2019). Studies reveal that integrating antimicrobial stewardship programs (ASPs) with infection prevention and control improves the efficacy of both approaches, as evidenced by notable decreases in infections, including *Clostridium difficile* infections, brought about by these coordinated initiatives (Knobloch et al., 2021). Moreover, in 2019, ICMR published the second version of its guidelines on using antibiotics in common syndromes, which includes updated information on how to treat skin infections, soft tissues, central nervous system, and bones and joints (Sandeep et al., 2020). So that ASPs are necessary for optimizing antibiotic use, improving patient safety, and lowering AMR (Ponto, 2010).

Antimicrobial stewardship methods can be applied in several ways:

#### 5.3.1 Educating and screening of patients

Before prescribing antibiotics, screening and interacting with patients can help to reduce needless antibiotic use in healthcare settings. This can be of help to establish whether antibiotics are truly needed in a particular case. The Centers for Disease Control and Prevention have released a useful checklist to guide the healthcare facilities in more efficiently implementing the practice of screening patients for antibiotics (Sanchez, 2016).

#### 5.3.2 Policy update

Updating hospital policy regarding antimicrobial stewardship initiatives is one of the most effective ways to enhance patient care and reduce needless medication use (Walia et al., 2019).

#### 5.3.3 Limiting the use of antibiotics as a prevention strategy

The primary aim of antibiotics is to treat infections, not to prevent. However, there are some circumstances where antibiotics can be used for prophylaxis. Because of spread of AMR in hospitals, many institutions have begun to use antibiotics as a last resort rather than a first line of defense because there are other general prophylaxis such as hand hygiene which can help stop infections even before they start (Andersen, 2019).

Centers for Disease Control and Prevention (CDC) has list of steps that should be followed by healthcare professionals to ensure proper hand hygiene: Ensure that hand hygiene supplies are always available to all healthcare professionals. Antibacterial soap or 2% chlorhexidine gluconate solution is also a better option for proper hand hygiene. However, any ethanol-based hand rub (70% v/v) will also be equally effective (Lemmen and Lewalter, 2018).

Studies have shown that antimicrobial stewardship interventions in outpatient settings, particularly those involving pharmacist-led actions based on microbiology culture reports, yield significant clinical benefits. In a study by Wattengel et al. (2020), acceptance of pharmacist recommendations was associated with markedly lower rates of 30-day treatment failure (5% *vs* 28%) and 30-day hospital admission (0.7% *vs* 11%) compared with cases where the interventions were rejected.

#### 5.3.4 De-escalation/modification

Modify empiric broad spectrum antibiotics depending on culture and antimicrobial susceptibility reports and patient status. Avoid double or redundant gram negative or anaerobic coverage - Discontinue antibiotics if a non-infectious mimic identified - De-escalate combination therapy to a single agent (Aggarwal et al., 2019).

#### 5.3.5 Monitoring the path of every medication

Errors are expected whenever a drug is given to the patients. So, it is very crucial to track the path of every medication from the time it is prescribed until it is given to the patient. Maintaining records of medicine orders and administration is very crucial for tracking the path of medication order (Bankar et al., 2022).

#### 5.3.6 Staff education

Limiting the use of antibiotics is only one component of effective antimicrobial stewardship. But it also includes giving training to staff on alternative treatment methods such as the use of gloves and hand sanitizer to prevent the spread of infections. In addition, it involves educating staffs about how infections are spread generally (Pereira et al., 2017).

By implementing these methods, infections can be reduced which will reduce the overall requirement of medicines.

## 6 Conclusion and future prospective

This narrative review provides an overview of the AMR status in Indian healthcare settings, with an emphasis on outpatient settings. We explore the impact of AMR on clinical outcomes and review the current efforts and interventions such as AMR Stewardship programs. In addition, we comprehensively discussed precision medicine, pharmacogenomics and other Omics based approaches aimed at curbing AMR. This study highlights the growing issue of antimicrobial resistance (AMR), particularly from multidrug-resistant (MDR) Gram-negative bacteria, which presents serious difficulties for healthcare systems. MDR in common pathogens like *E. coli, Klebsiella pneumoniae*, and *Staphylococcus aureus* is well-documented, but specific resistance mechanisms remain underexplored. AMR studies often reflect data from selected regions in India or isolated hospitals. A research gap exists in understanding how resistance patterns vary across rural *vs*. urban settings and in primary, secondary, and tertiary healthcare levels.

Few studies assess the direct impact of AMR on treatment outcomes, length of hospital stay, and mortality rates. Poor sanitation, inappropriate antibiotic usage in agriculture, and a lack of healthcare infrastructure in rural regions are examples of environmental variables that contribute to the spread of AMR, which are poorly understood. Research focusing on the clinical effects of drug-resistant infections in vulnerable populations, particularly in Indian settings, utilizes pharmacogenomics methods to provide personalized treatments through genetic profiling and therapeutic drug monitoring. Additionally, studies on the effectiveness of antimicrobial stewardship initiatives and community-based ASPs aim to reduce antimicrobial resistance. Our knowledge of the elements contributing to resistance in developing nations such as India would be improved further by research integrating environmental and socioeconomic factors with AMR patterns.

The emergence of multidrug-resistant organisms (MDROs) raises morbidity and death rates which complicates treatment regimens and raises healthcare expenses from a clinical standpoint (Nathwani et al., 2014; Kadam et al., 2024). Treatment of infections caused by resistant pathogens, including extensively carbapenem-resistant *Enterobacterales* (CRE), and methicillin-resistant *Staphylococcus aureus* (MRSA), is difficult because only few effective antibiotics are available. When pan-resistant strains appear, it is then especially concerning and highlights the seriousness of the issue because for that there are now no viable antibiotics.

Using antibiotics carelessly in aquaculture and agriculture has made the issue worse. The widespread use of antibiotics for both infection treatment and livestock growth promotion lead to the selection of resistant strains that can spread to humans through the food chain and environmental pathways (Page and Gautier, 2012). The necessity of a “One Health” approach that acknowledges the interdependence of environmental, animal, and human health is highlighted (Jani et al., 2021). Antimicrobial stewardship programs (ASPs) are essential for reducing AMR. Targeted therapy, which lessens the need for broad-spectrum antibiotics and the selection pressure for resistant bacteria, can be made possible by quick and precise diagnostic methods. In the same way, ASPs urge for careful consumption of antibiotics, supporting de-escalation techniques, suitable dosage, and treatment length to curb antimicrobial resistance.

The pharmaceutical pathway for novel antibiotics is still limited due to scientific, regulatory and financial obstacles. Developing new antibiotics requires modest commercial returns, significant time, investment and commitment. By promoting research in novel anti-infective therapy, we can revive the antimicrobial pipeline for which we would need development through public private collaborations, market entry incentives, simplified regulatory processes and international cooperation which is essential to effectively address AMR. International frameworks like World Health Organization (WHO) have goals on Global Action Plan (GAP) on AMR which are, are stronger surveillance, better infection prevention and control and easier access to high-quality antimicrobials. However, there have been major obstacles such as antimicrobial access and healthcare infrastructure of some nations.

A successful response towards curbing AMR requires addressing these inconsistencies through technology transfer, capacity building and fair access to diagnostics and antimicrobials. Precision medicine helps to make customized and informed medical decisions, tailoring therapies by considering patient’s genetic makeup, immune response, specific infection, and socio-environmental and lifestyle factors. By using precision medicine’s patient-centric approach we can closely monitor drug levels using therapeutic drug monitoring (TDM) (Roberts et al., 2012). This helps maintain the peak therapeutic efficacy of drugs while mitigating adverse effects and drug-related toxicities. In conclusion, we need more research focusing on the clinical consequences of drug-resistant infections in vulnerable populations especially in Indian Outpatient settings which could be achieved using pharmacogenomics methods to provide personalized treatments using genetic profiling and TDM.

## Glossary

ABCB1: ATP-Binding Cassette Subfamily B Member 1, ABCB1, ATP-Binding Cassette Subfamily B Member 1
ABCC2: ATP-Binding Cassette Subfamily C Member 2
ABCC4: ATP-Binding Cassette Subfamily C Member 4
ABCG2: ATP-Binding Cassette Subfamily G Member 2
ABCG2: ATP-Binding Cassette Subfamily G Member 2
AMR: Antimicrobial resistance
AMRSN: AMR Monitoring Network
ARGs: Antimicrobial Resistance Genes
CAP: Community Acquired Pneumonia
CRE: Carbapenem-resistant *Enterobacterales*
DILI: Drug Induced Liver Injury
DRESS: Drug Reaction with Eosinophilia and Systemic Symptoms
FDCs: Fixed Drug Combinations
GCLC: Glutamate-Cysteine Ligase Catalytic Subunit
HAIs: Hospital-acquired Infections
ICMR: Indian Council of Medical Research
LRTIs: Lower Respiratory Tract Infections
MDR-GNB: Multi-drug-resistant Gram-negative Bacteria
MIC: Minimum Inhibitory Concentration
MRSA: Methicillin-Resistant *Staphylococcus aureus*
NAP-AMR: National Action Plan on Antimicrobial Resistance
NARS-Net: National Antimicrobial Resistance Surveillance Network
NCDC: National Centre for Disease Control
OTC: Over-the-counter
PTPN22: Protein Tyrosine Phosphatase, Non-Receptor Type 22
PXR: Pregnane X Receptor
SLC22A8: Solute Carrier Family 22 Member 8
SLCO1B1: Solute Carrier Organic Anion Transporter Family Member 1B1
SLCO1B1: Solute Carrier Organic Anion Transporter Family Member 1B1
SSTIs: Skin & Soft Tissue Infections
UGT1A: UDP glucuronosylytransferase family 1
UTIs: Urinary Tract Infection
VRE: Vancomycin-Resistant *Enterococcus*
WHO: World Health Organization
WHONET: World Health Organization Network
